# Supplementary figures and images for: Why do G-quadruplexes dimerize through the 5’-ends? Driving forces for G4 DNA dimerization examined in atomic detail
Source: PLoS Comput Biol. 2019 Sep 20;15(9):e1007383. doi: 10.1371/journal.pcbi.1007383 (PMC6774569; doi:10.1371/journal.pcbi.1007383)

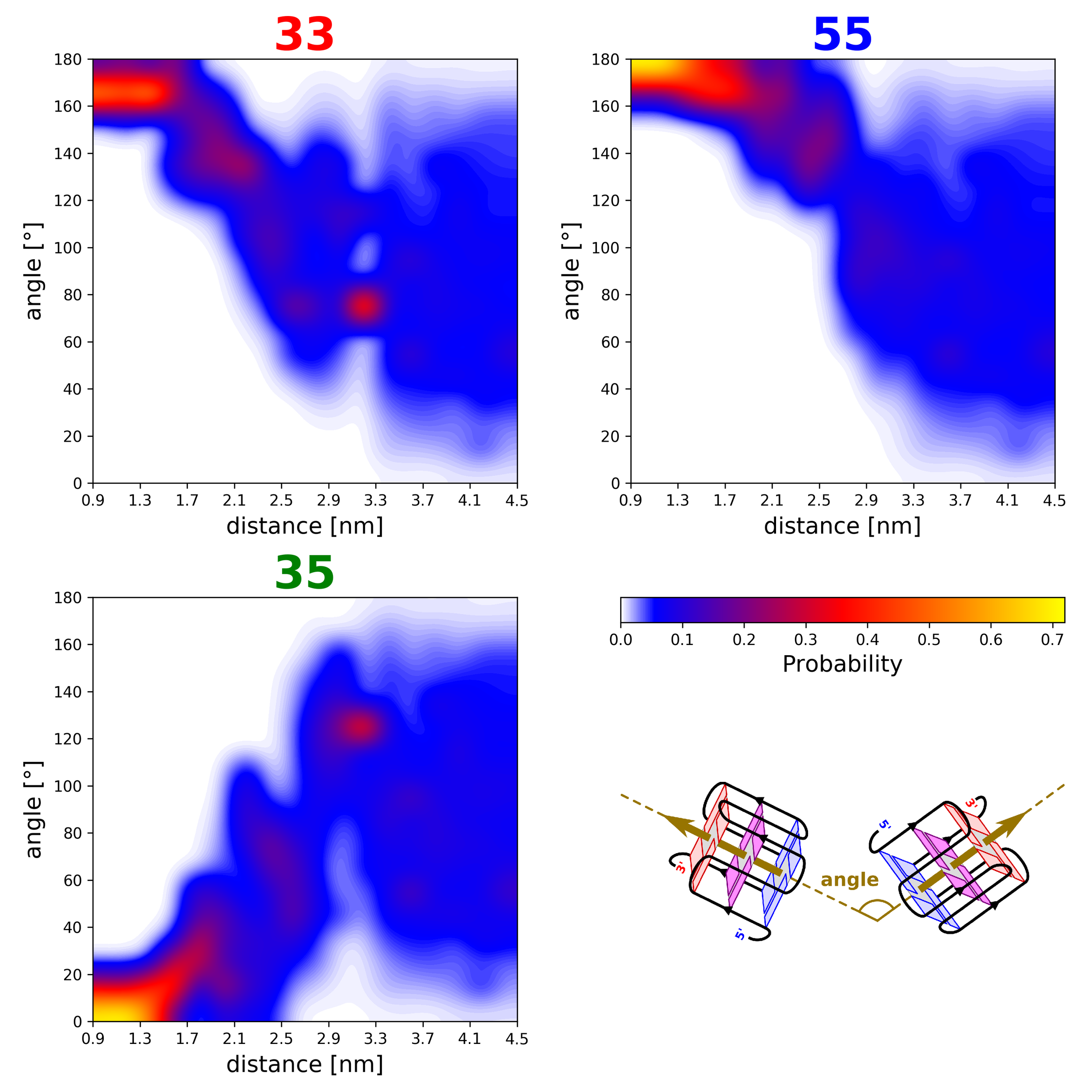

Supplement: S1 Fig — The orientation angle between G4 units is defined as the angle between two vectors passing through the center of G-tetrads and perpendicular to them. It can be seen that G-quadruplexes dimerize through cofacial stacking (parallel orientation between the interfacial G-tetrads at short distances, with the orientation angle ≈ 180° or 0° for 5-5 and 3-3 or 3-5 dimers, respetively). At longer distances the systems sample the entire range of relative orientations between G-quadruplexes (the orientation angle in the range of 0–180°). (TIF) [file pcbi.1007383.s001.tif]

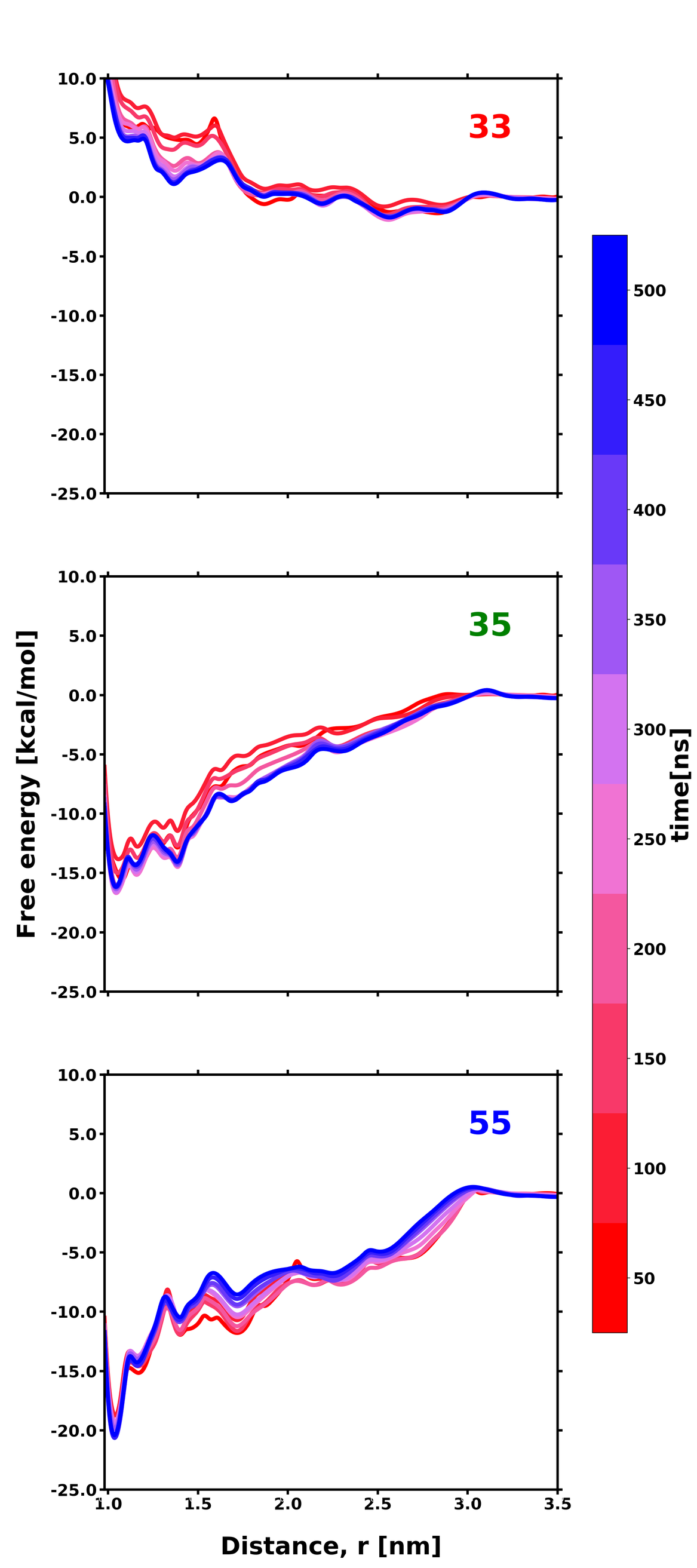

Supplement: S2 Fig — (TIF) [file pcbi.1007383.s002.tif]

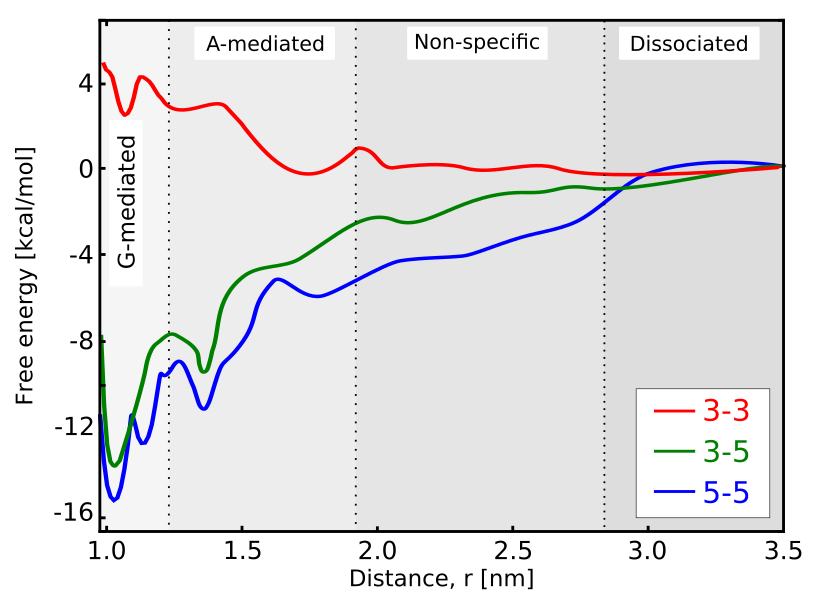

Supplement: S3 Fig — Free energy profiles for the formation of the dimers computed using parmbsc1 as a function of the separation distance between them, r. Even though dimer formation is consistently less favorable than in the CHARMM36 simulations, the stacking preferences are the same. (TIF) [file pcbi.1007383.s003.tif]

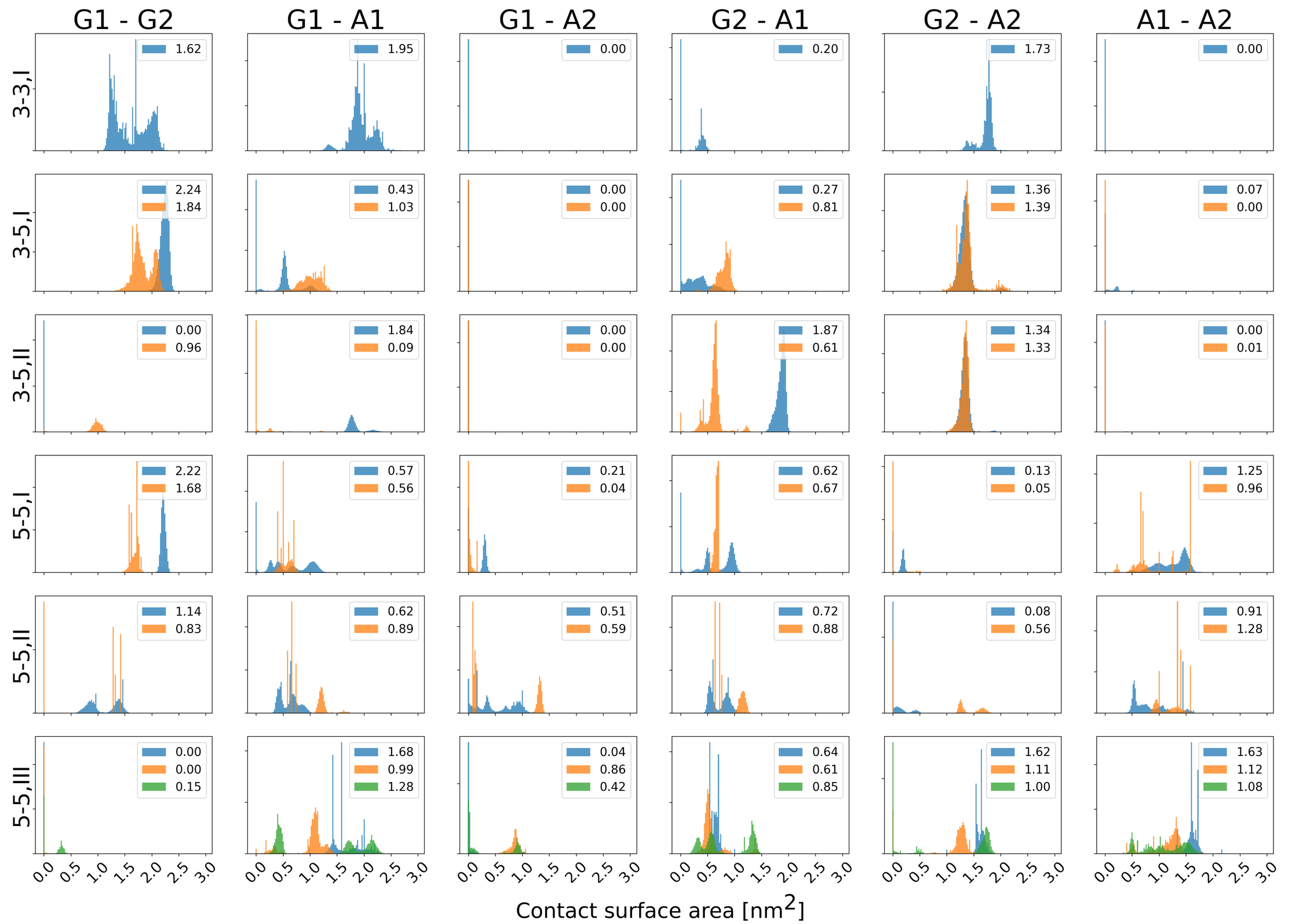

Supplement: S4 Fig — Different colors are used to map the distributions on the structural clusters produced by our cluster analysis. (TIF) [file pcbi.1007383.s004.tif]

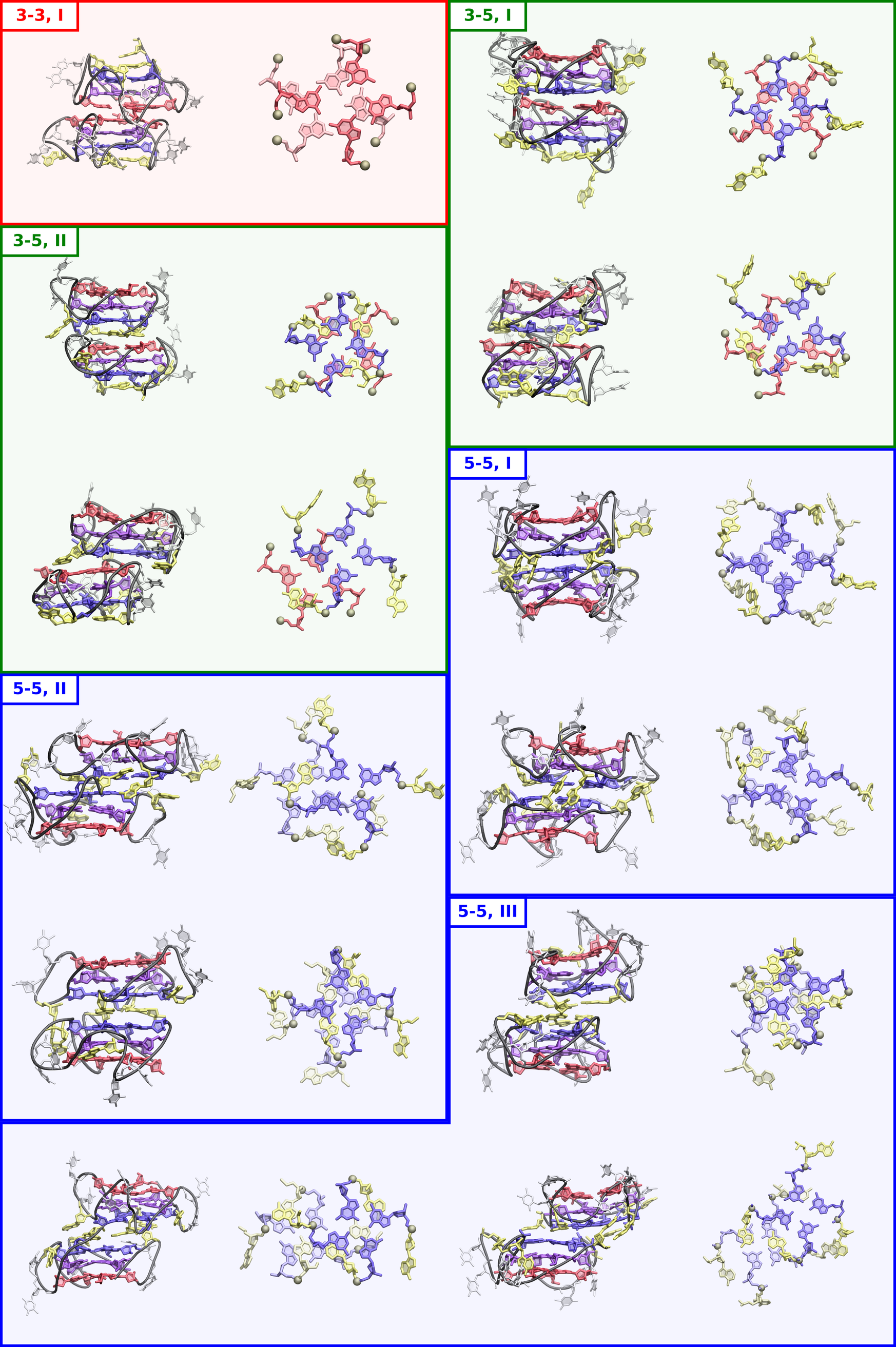

Supplement: S5 Fig — (TIF) [file pcbi.1007383.s005.tif]

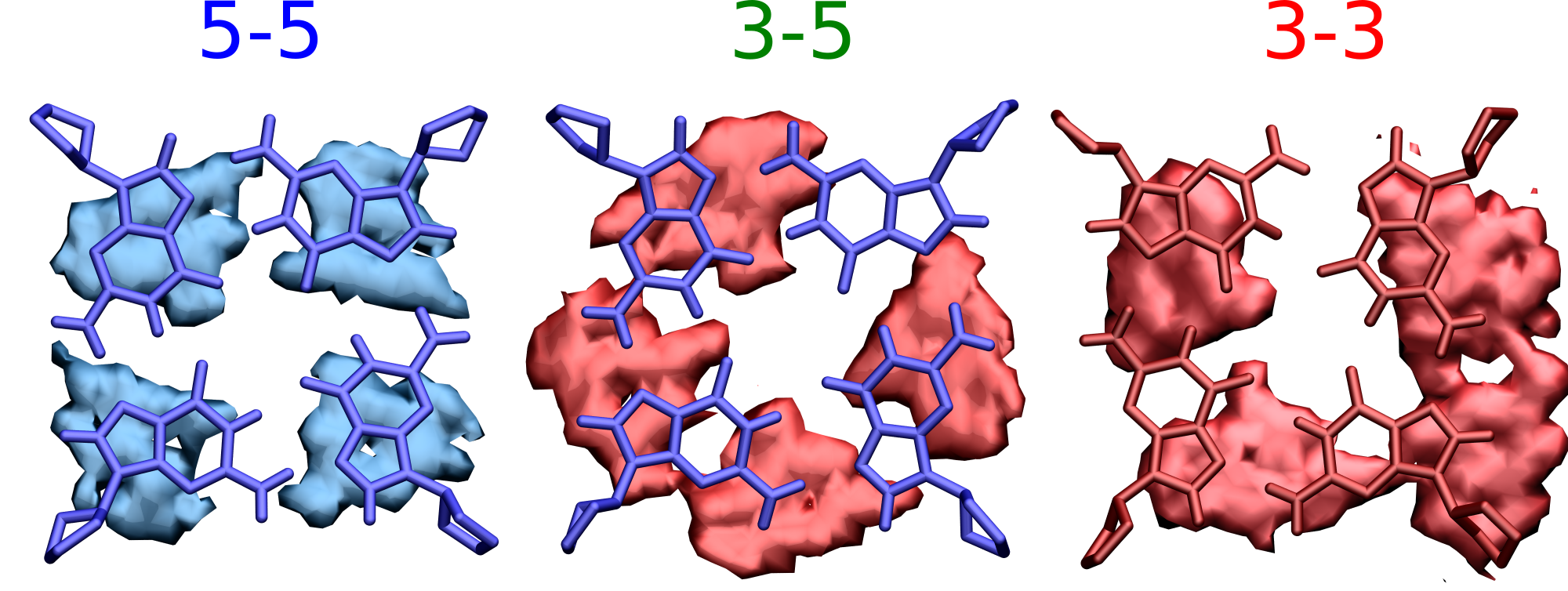

Supplement: S6 Fig — (TIF) [file pcbi.1007383.s006.tif]

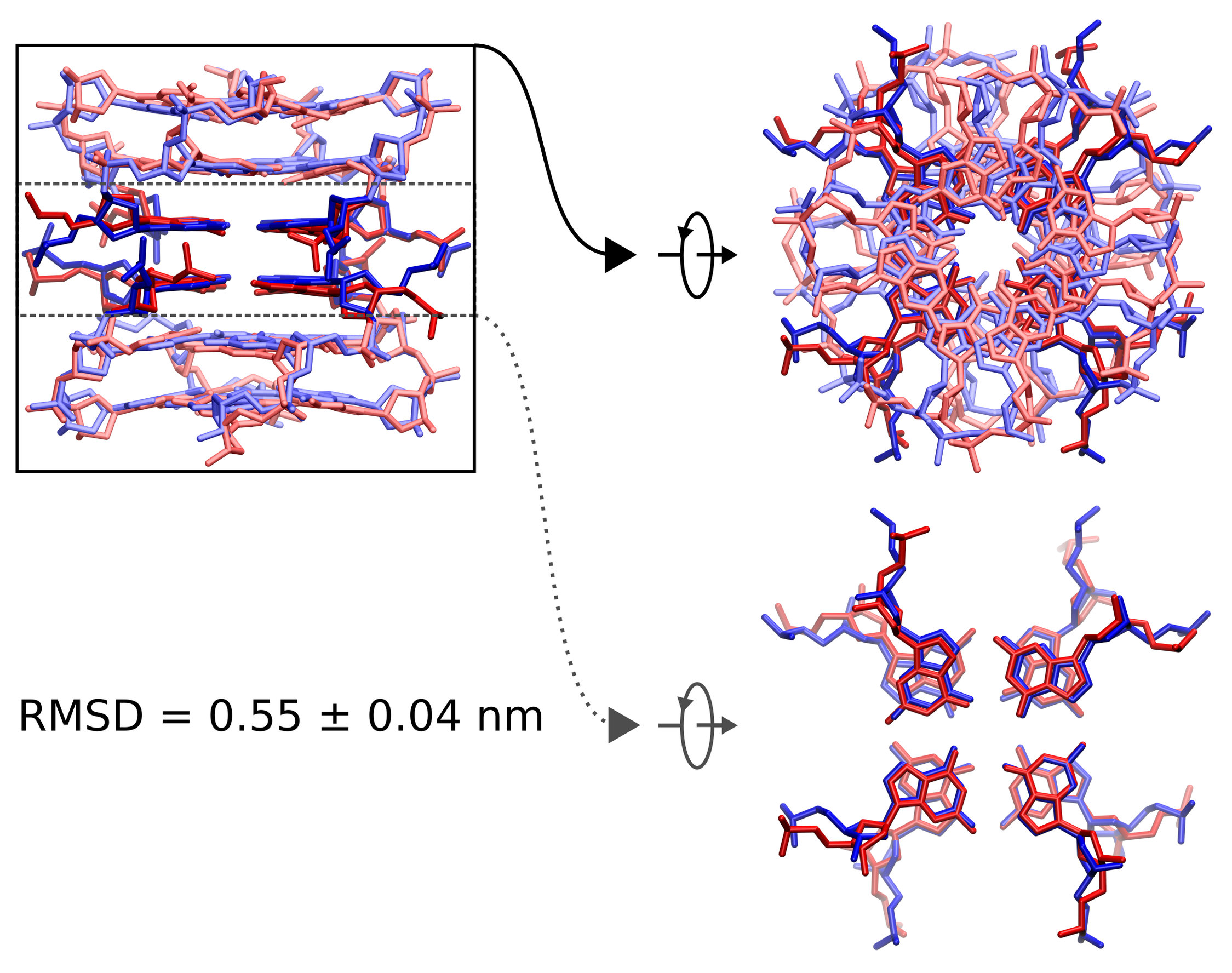

Supplement: S7 Fig — Structural comparison of the 5-5 dimer predicted by our MD simulations (red, 5-5,I in Fig 1B) with the 5-5 stacking arrangement of parallel telomeric G-quadruplexes found in the x-ray structure (blue, PDB: 1KF1). The structures were superimposed based on the positions of the phosphate groups. The average heavy-atom RMSD between the MD snapshots and the x-ray structure is 0.55 nm. (TIF) [file pcbi.1007383.s007.tif]

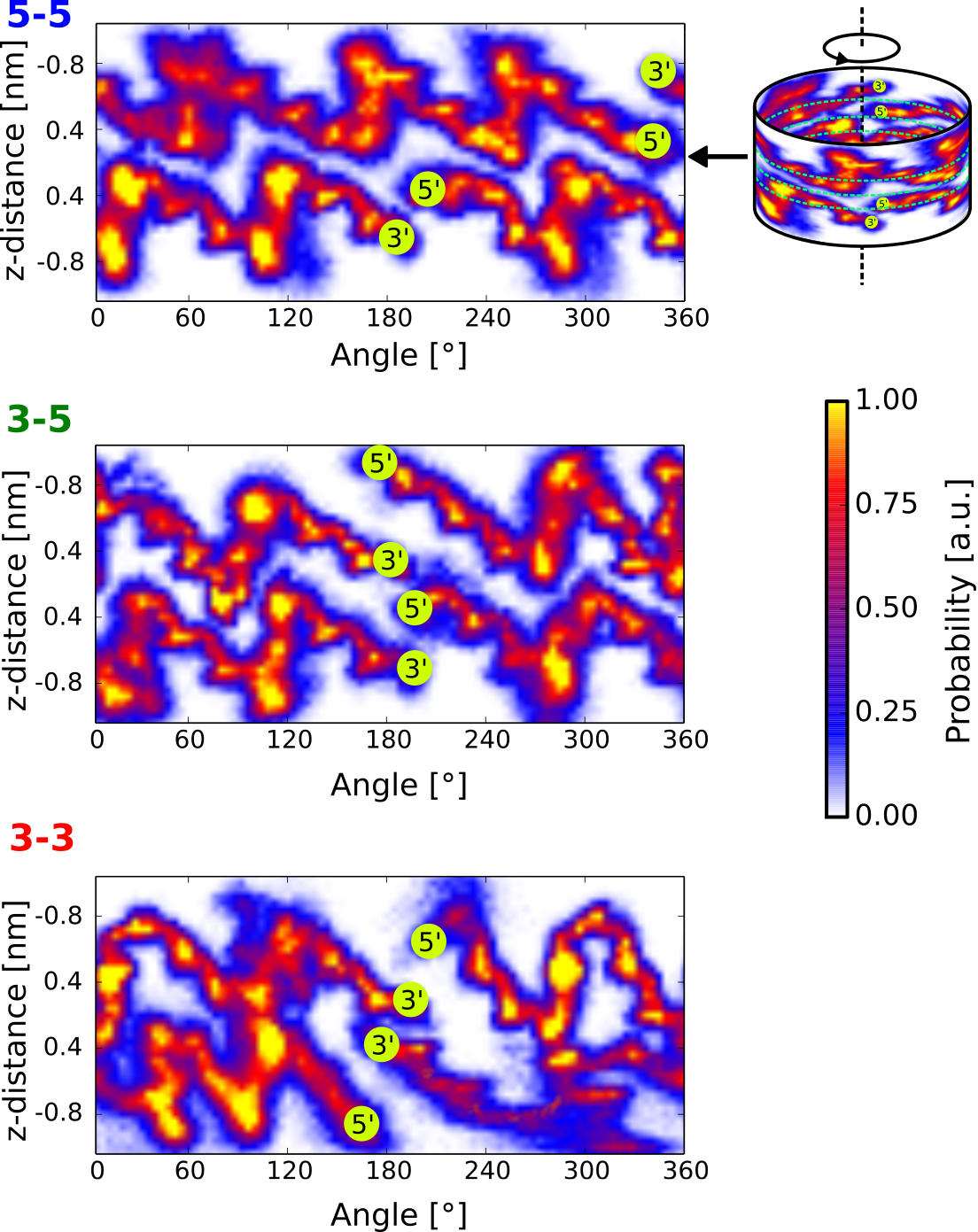

Supplement: S8 Fig — Relative arrangement presented as projections of the atomic positions of backbones on the curved surface of the cylinder with a diameter corresponding to the G-quadruplex dimer. (TIF) [file pcbi.1007383.s008.tif]

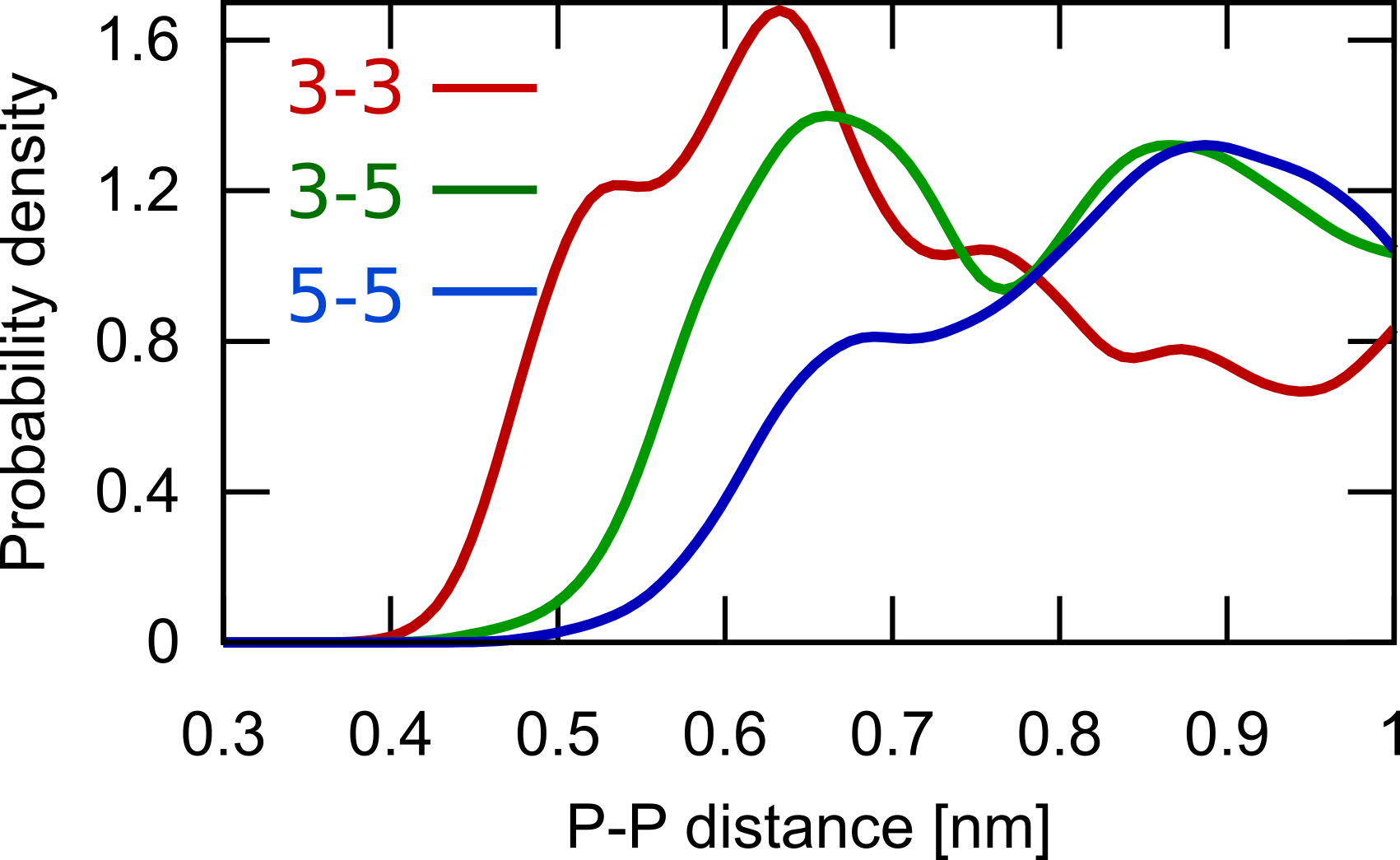

Supplement: S9 Fig — (TIF) [file pcbi.1007383.s009.tif]

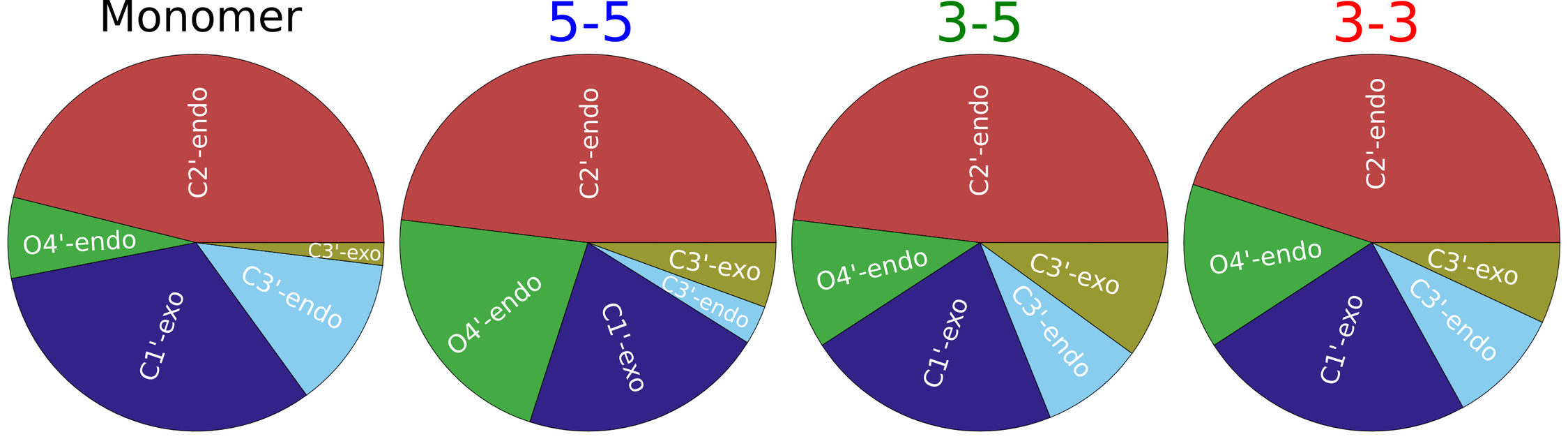

Supplement: S10 Fig — (TIF) [file pcbi.1007383.s010.tif]

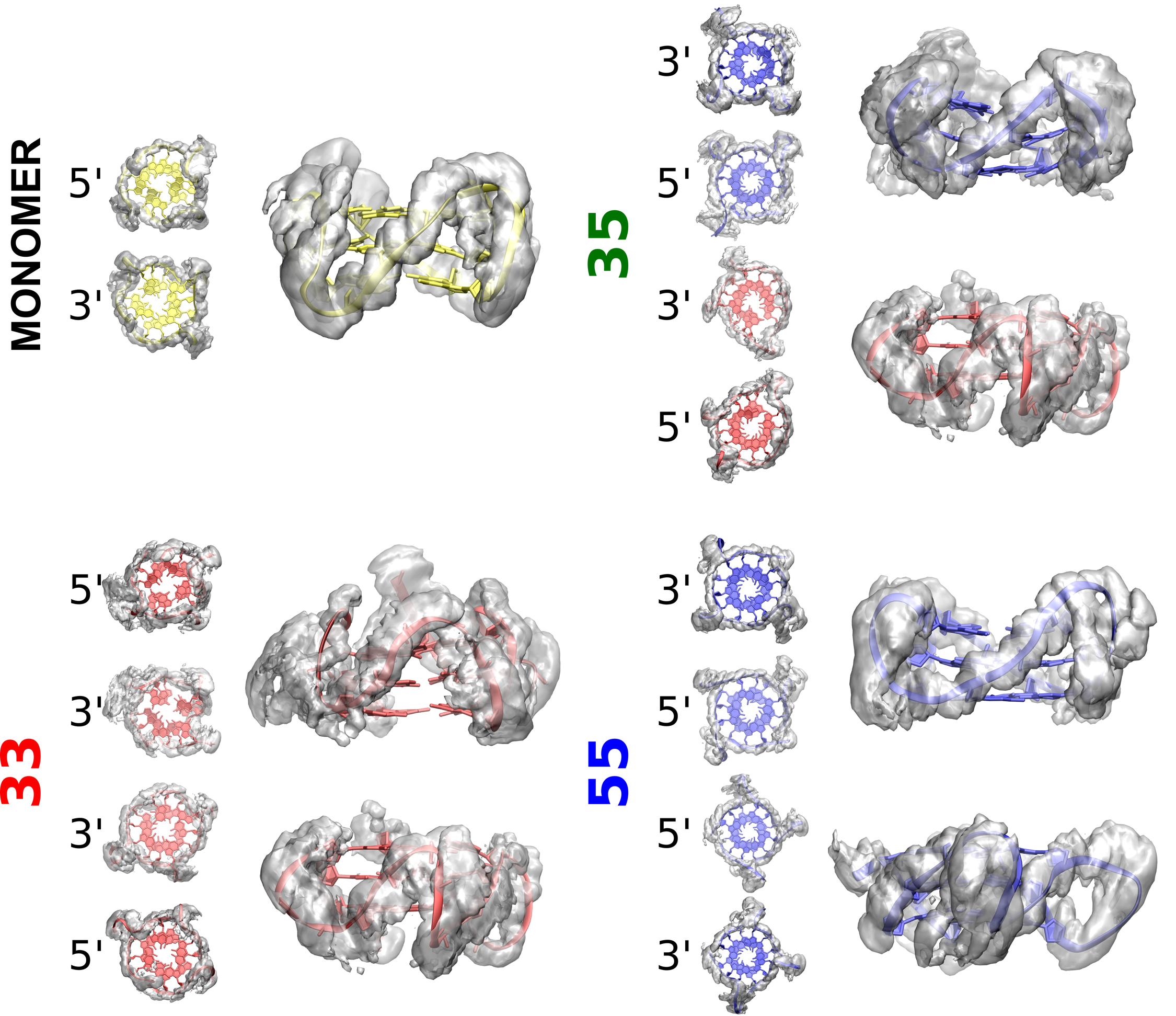

Supplement: S11 Fig — Isosurfaces show 90% of the total fluctuation range. For each G4 monomer one side and two top views (from the 5’- and 3’-ends) are shown. (TIF) [file pcbi.1007383.s011.tif]

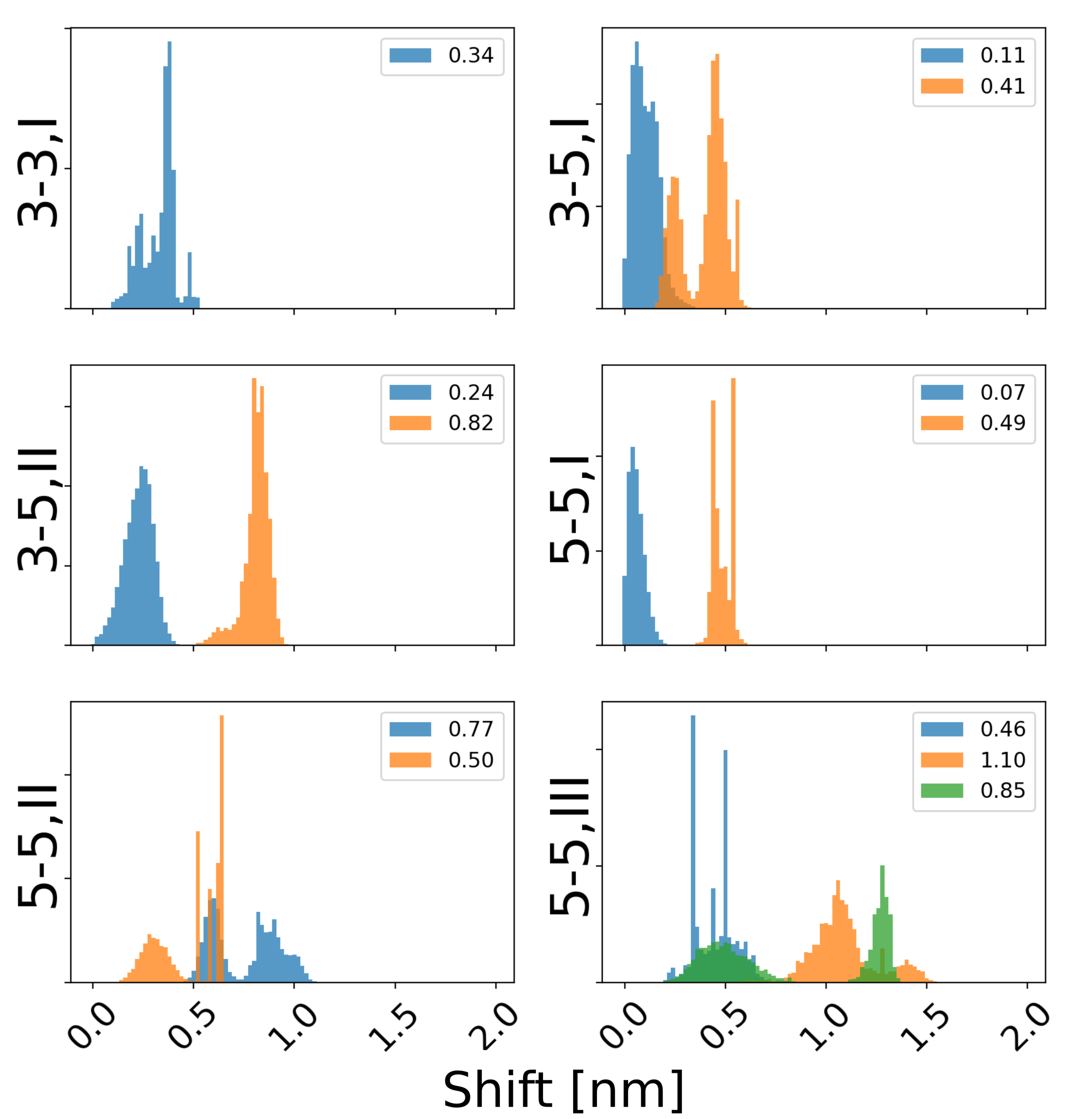

Supplement: S12 Fig — Shift is defined as the distance between the axes of the G4 units projected on the stacking interface plane. The axes were determined as vectors connecting the centers of mass of the two external G-tetrads of each of the G4 units. Different colors are used to map the distributions on the structural clusters produced by our cluster analysis. (TIF) [file pcbi.1007383.s012.tif]

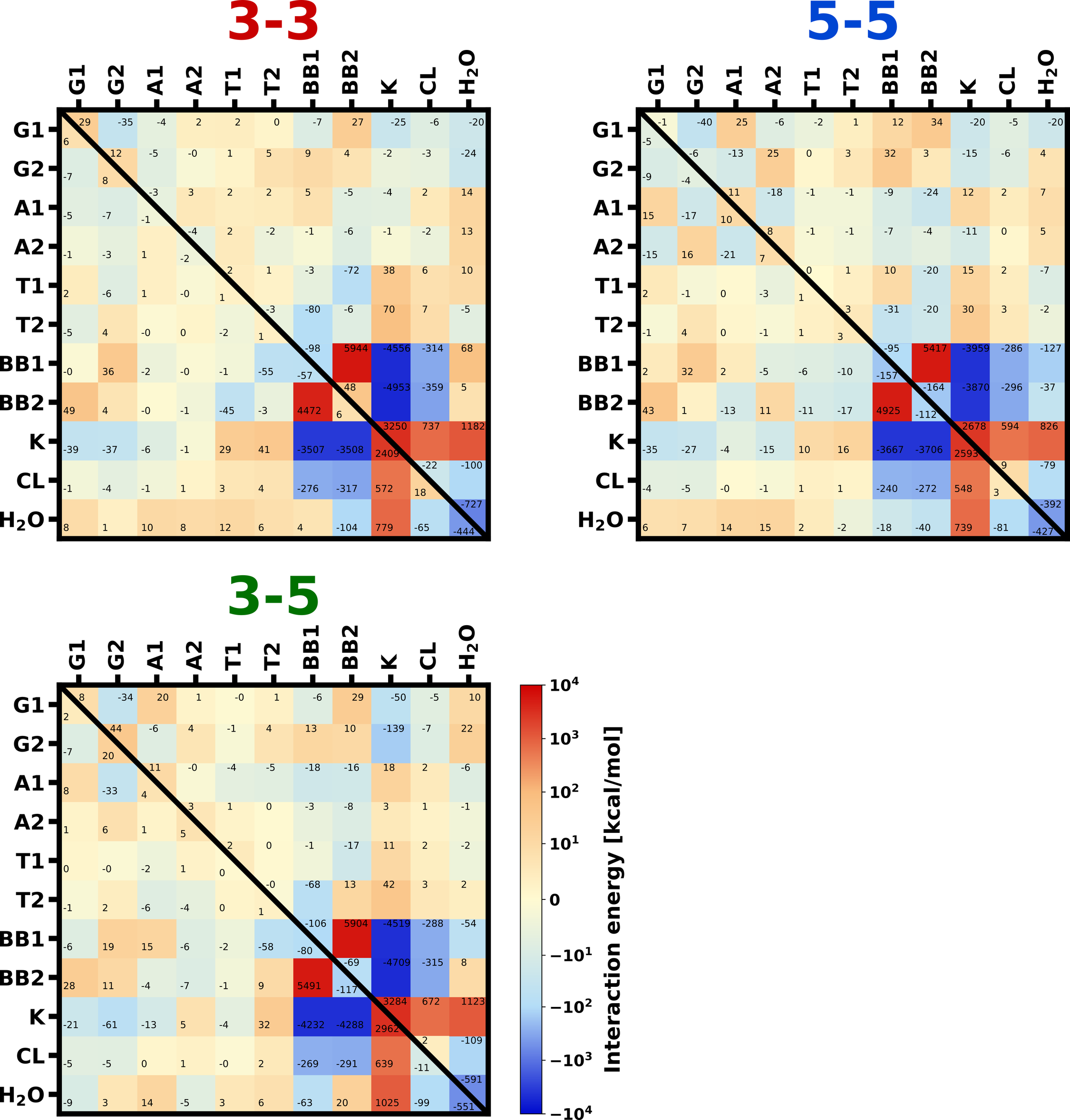

Supplement: S13 Fig — Detailed contributions to the dimerization enthalpy (ΔH) computed as the interaction energy changes between individual structural elements of the system: guanines (G), adenines (A), thymines (T), backbone (BB), potassium cations (K), chloride anions (CL) and water (H2O) in the G-mediated state (upper triangle matrix) and the A-mediated state (lower triangle matrix); individual G4 monomers are denoted as 1 and 2. For clarity, numeric values were rounded to the nearest integer. (TIF) [file pcbi.1007383.s013.tif]

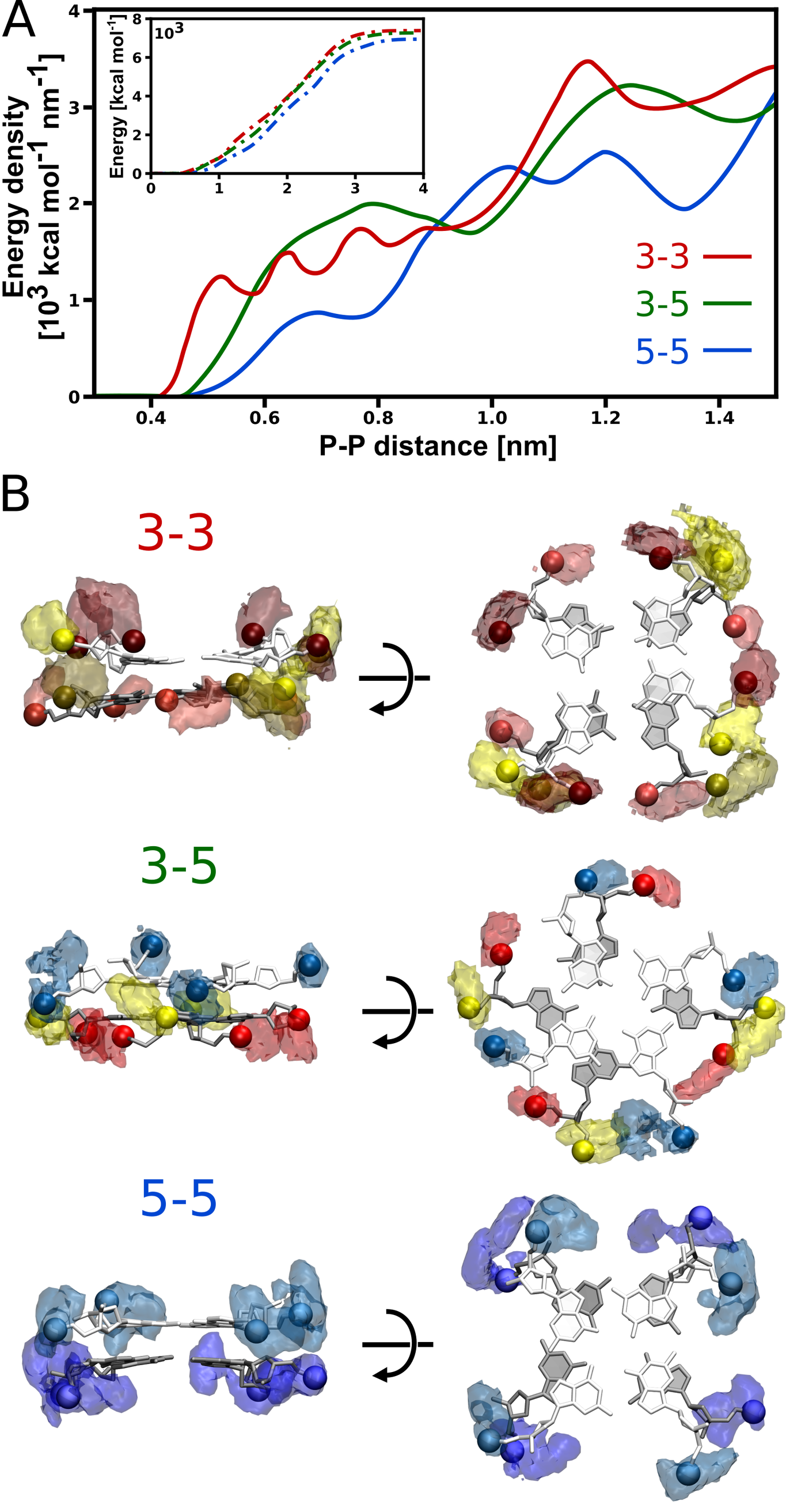

Supplement: S14 Fig — A) Electrostatic energy density of pairwise cross-strand interactions between the phosphate groups as a function of the distance between them in the parmbsc1 force field simulations. The inset shows cumulative interphosphate repulsion energy as a function of the P–P distance. B) Spatial distribution of all phosphate groups at the stacking interface that are located within 1 nm of any phosphate group of the other G4 monomer in the parmbsc1 force field simulations. The phosphate groups of the nucleotides in the 3’- and 5’-terminal G-tetrads are shown in red and blue, respectively, while the remaining ones in yellow. (TIF) [file pcbi.1007383.s014.tif]

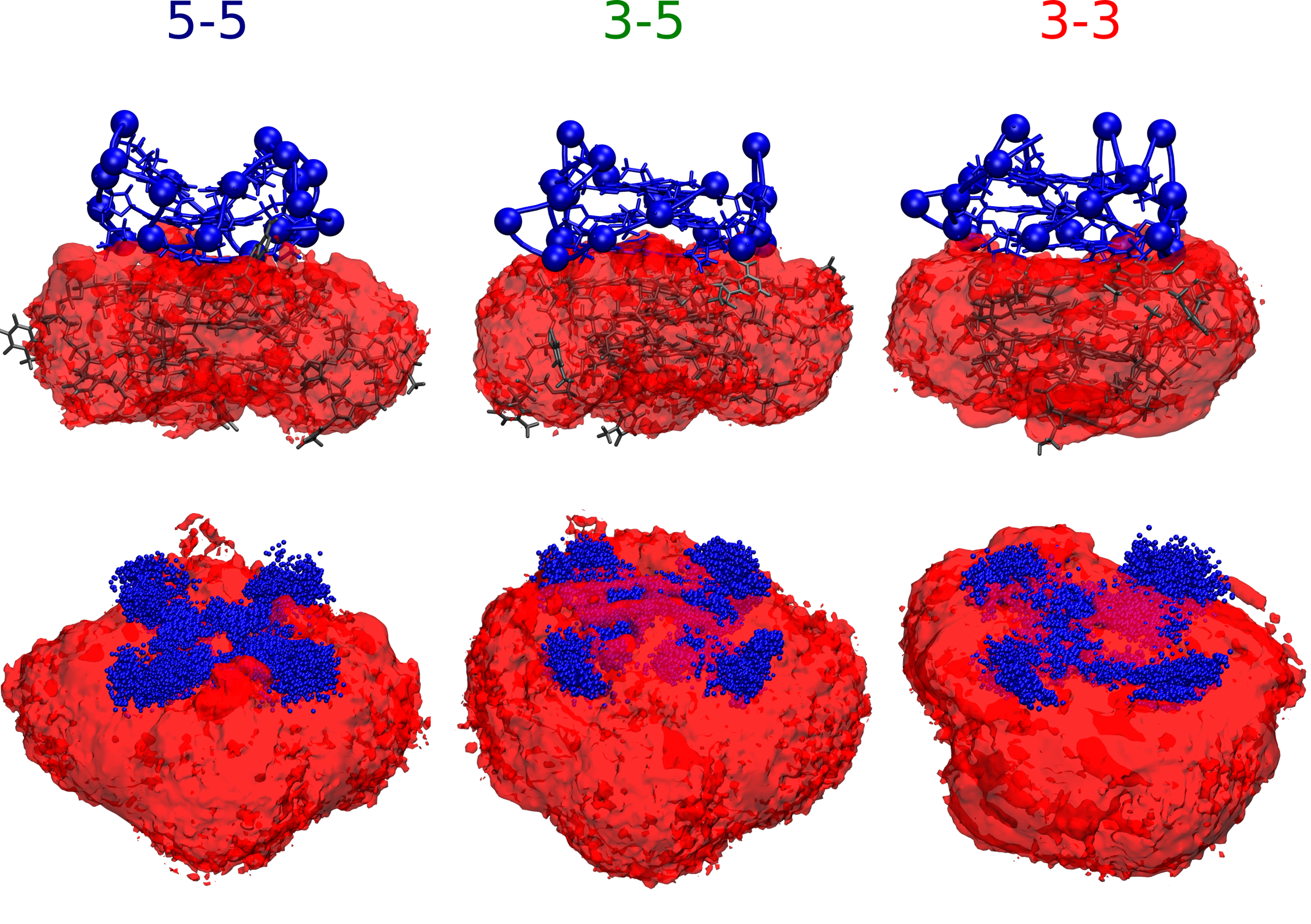

Supplement: S15 Fig — Distribution obtained by integrating the linearized Poisson Boltzmann equation using Adaptive Poisson-Boltzmann Solver (APBS) (N. A. Baker, et al. Proc. Natl. Acad. Sci. U.S.A. 98, 10037, 2001). The concentrations of +1 and −1 ion species were set to 150 mM with an ion exclusion radius of 0.2 nm. Grid size of 1203 with grid spacing of 0.069 nm was used. We used the Dirichlet boundary conditions with the boundary potential value determined from a Debye-Huckel model for a single sphere with a point charge, dipole, and quadruple. Blue balls in the top panel represent the positions of the phosphate groups of the opposite G4 unit. Blue dots in the bottom panel show the distribution of all heavy atoms in the adjacent guanine plane of the opposite G4 unit. (TIF) [file pcbi.1007383.s015.tif]

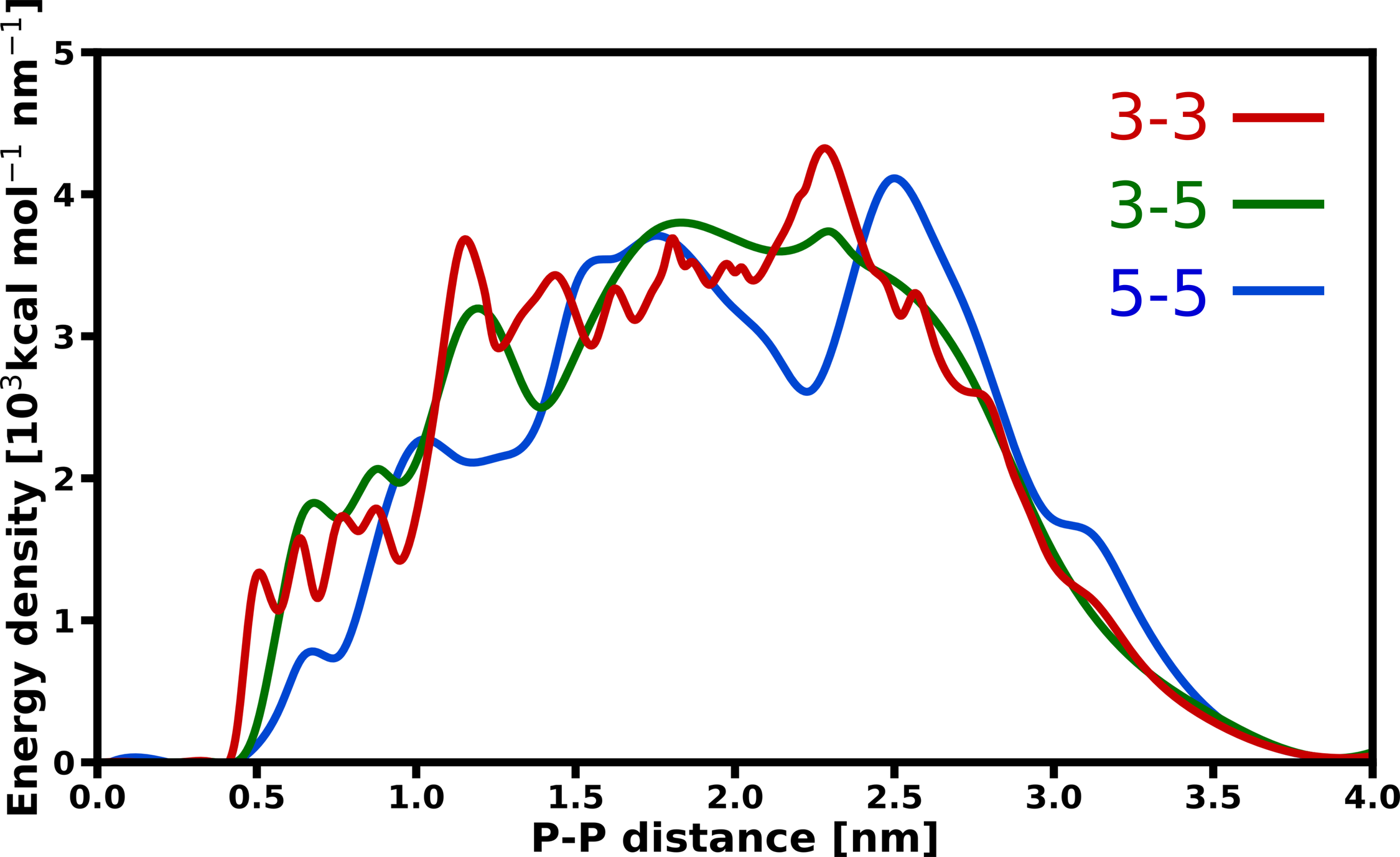

Supplement: S16 Fig — (TIF) [file pcbi.1007383.s016.tif]

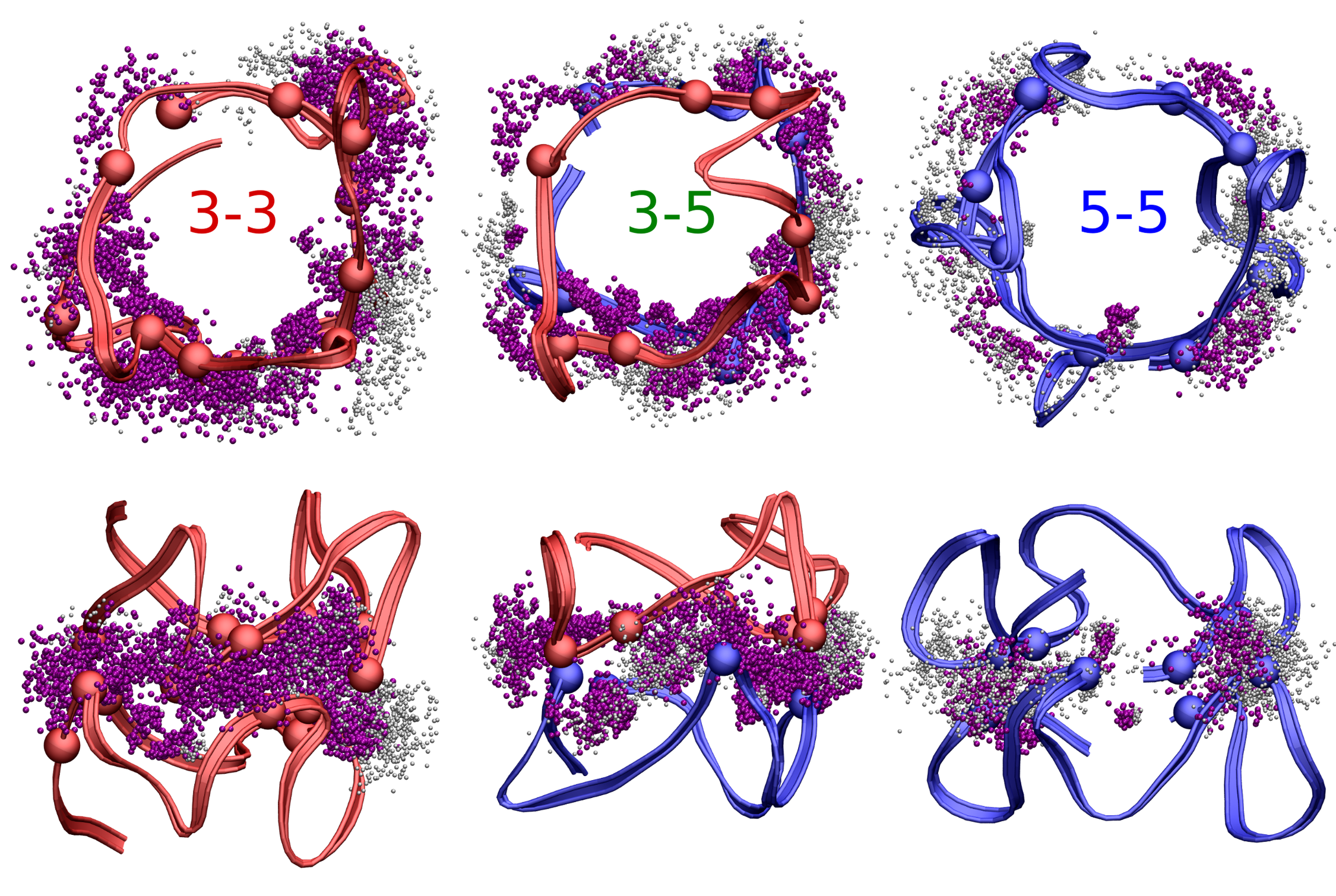

Supplement: S17 Fig — Generated by superimposing 1,000 0.5-ns-separated snapshots at a physiological KCl concentration from the parmbsc1 force field simulations. The interface K+ ions that at the same time are in contact with the PO4 groups of both G4 units are highlighted in purple. (TIF) [file pcbi.1007383.s017.tif]

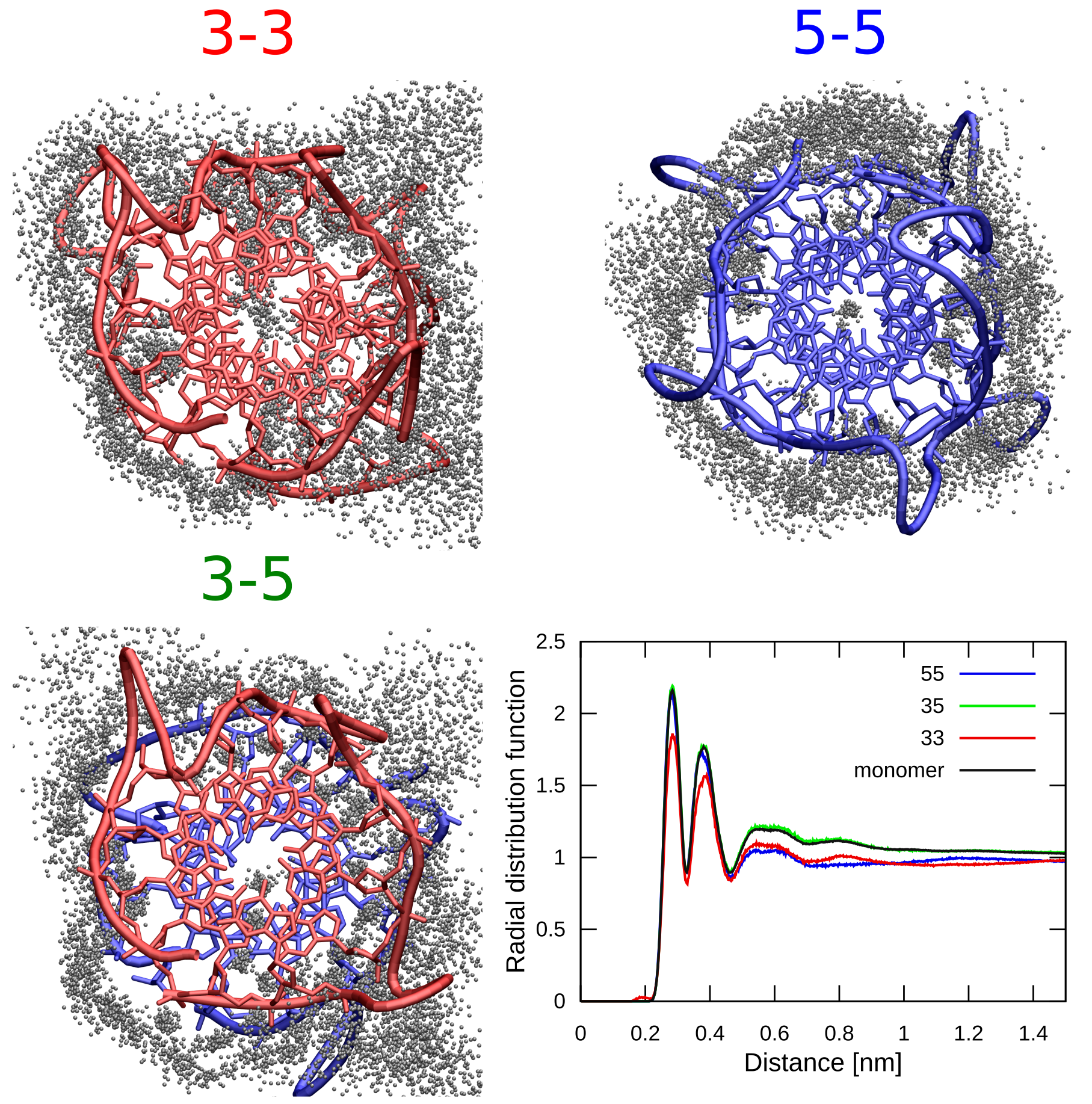

Supplement: S18 Fig — Generated by superimposing 100 5-ns-separated snapshots at a physiological KCl concentration. The plot shows radial distribution functions of water molecules with respect to the phosphate groups of the nucleotides in the external G-tetrads (monomer) or the G-tetrads involved in dimer formation (dimers). (TIF) [file pcbi.1007383.s018.tif]

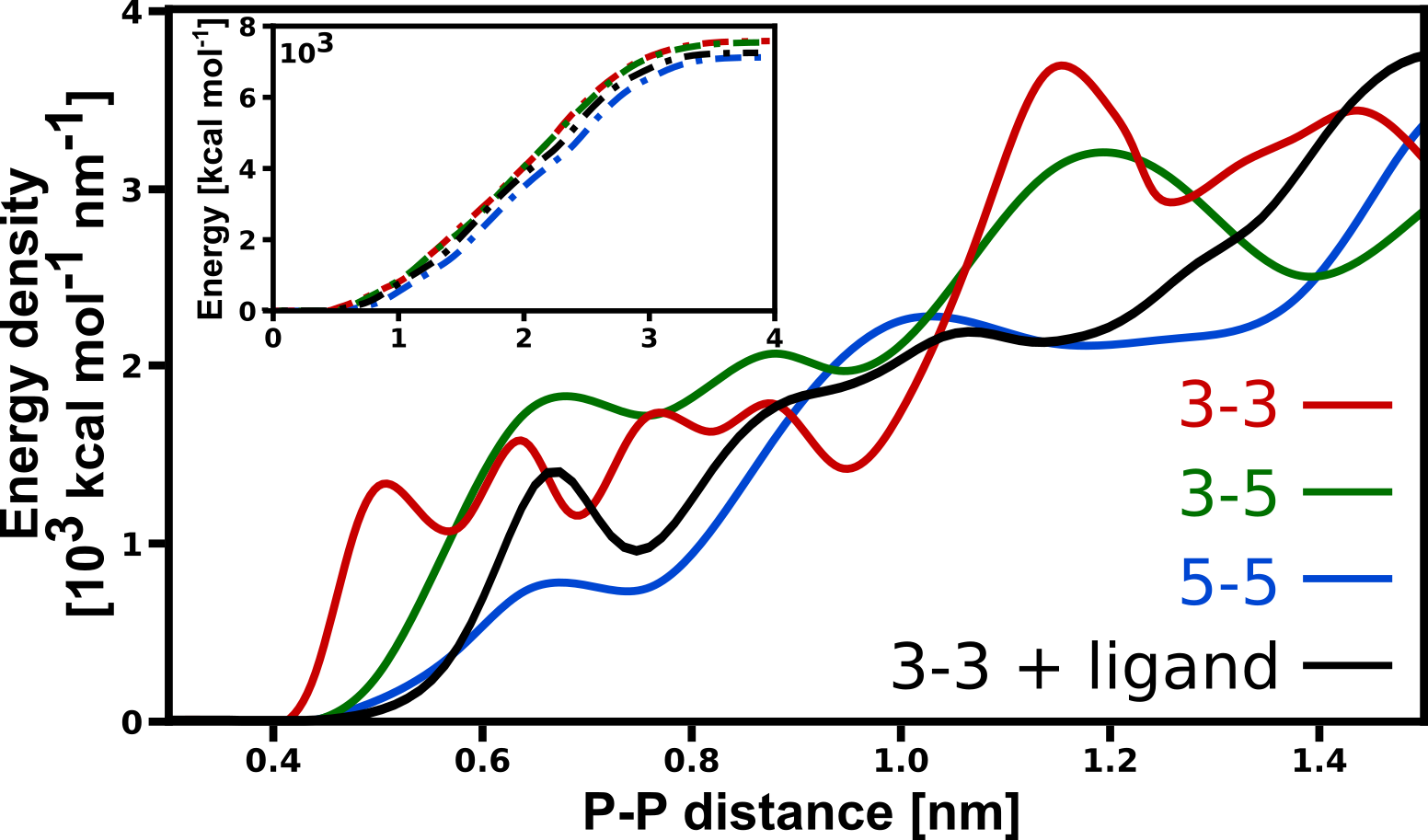

Supplement: S19 Fig — Effect of the 3,4-TMPyPz ligand intercalated between the two G4 units on the electrostatic energy density of pairwise cross-strand interactions between the phosphates as a function of the distance between them (P–P distance). The inset shows cumulative interphosphate repulsion energy as a function of the P–P distance. (TIF) [file pcbi.1007383.s019.tif]

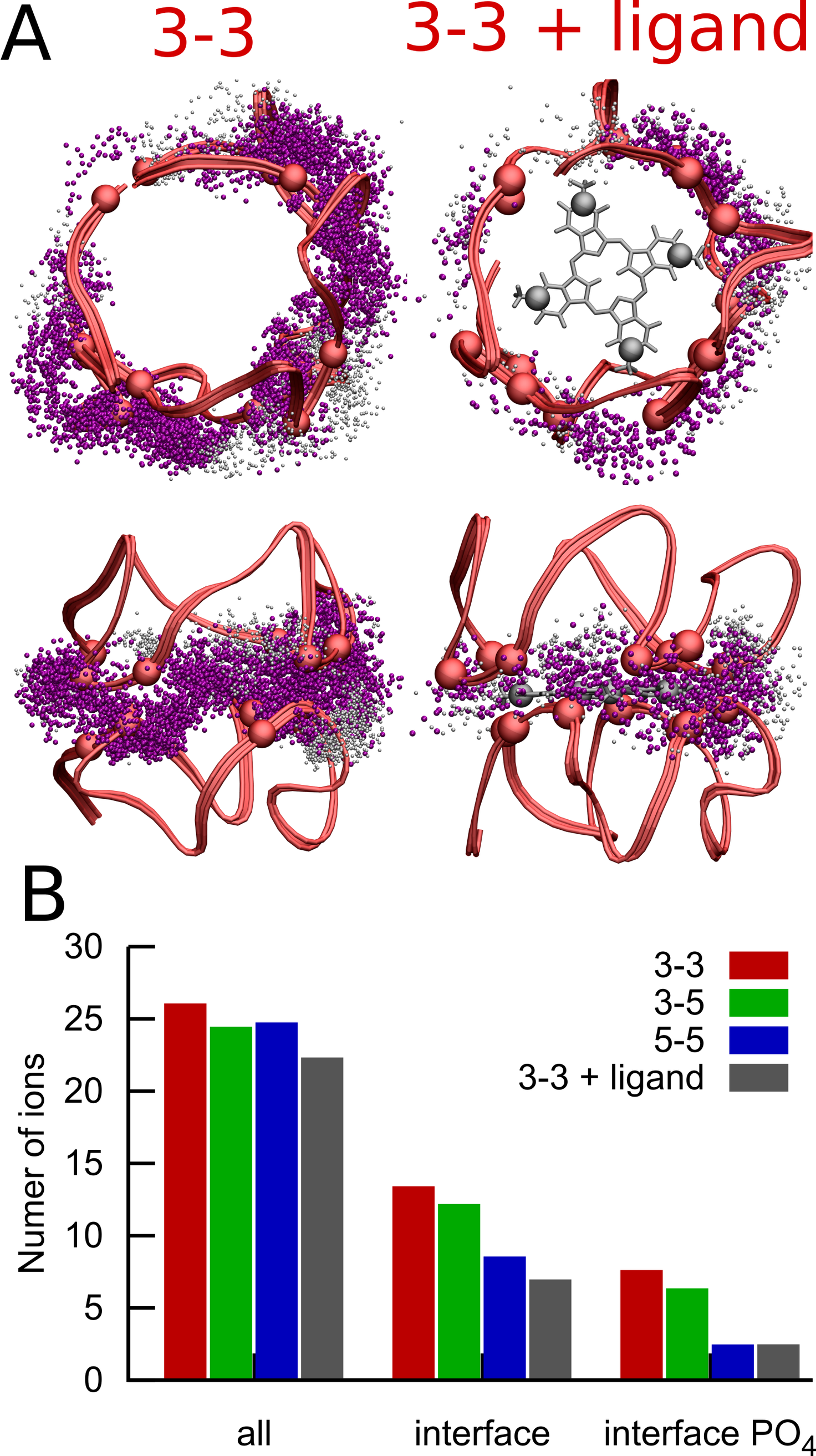

Supplement: S20 Fig — A) The images were generated by superimposing 1,000 0.5-ns-separated snapshots at a physiological KCl concentration. The interface K+ ions that at the same time are in contact with the PO4 groups of both G4 units are highlighted in purple. B) Average numbers of K+ ions in contact with the G-mediated dimers: within 0.7 nm of the backbones of any G4 unit (all), within 0.7 nm of both backbones at the same time (interface), within 0.7 nm of the PO4 groups of the two G4 units at the same time (interface PO4). (TIF) [file pcbi.1007383.s020.tif]
