# Supplementary material for: Why do G-quadruplexes dimerize through the 5’-ends? Driving forces for G4 DNA dimerization examined in atomic detail
Source: PLoS Comput Biol. 2019 Sep 20;15(9):e1007383. doi: 10.1371/journal.pcbi.1007383 (PMC6774569; doi:10.1371/journal.pcbi.1007383)

S1 Table: Partial charges obtained for 3,4-TMPyPz ligand.

index	atom type	charge	index	atom type	charge	index	atom type	charge	index	atom type	charge
1	C	0.026	21	C	0.167	41	C	-0.221	61	H	0.191
2	N	0.087	22	N	0.087	42	C	0.104	62	H	0.191
3	C	0.167	23	C	0.104	43	N	0.087	63	C	-0.336
4	C	-0.294	24	C	-0.221	44	C	0.167	64	H	0.191
5	C	0.084	25	N	-0.715	45	C	0.167	65	H	0.191
6	C	-0.221	26	H	0.202	46	N	0.087	66	H	0.191
7	C	0.688	27	H	0.222	47	C	0.026	67	C	-0.336
8	N	-0.604	28	H	0.172	48	C	-0.221	68	H	0.191
9	C	0.777	29	C	0.688	49	H	0.172	69	H	0.191
10	H	0.172	30	N	-0.604	50	H	0.222	70	H	0.191
11	H	0.222	31	C	0.777	51	H	0.202			
12	H	0.202	32	C	-0.294	52	H	0.172			
13	H	0.371	33	C	0.084	53	H	0.202			
14	N	-0.715	34	C	0.777	54	H	0.222			
15	N	-0.715	35	N	-0.709	55	C	-0.336			
16	C	0.688	36	C	-0.294	56	H	0.191			
17	N	-0.709	37	C	0.084	57	H	0.191			
18	C	0.777	38	C	0.688	58	H	0.191			
19	C	-0.294	39	N	-0.715	59	C	-0.336			
20	C	0.084	40	C	0.371	60	H	0.191			

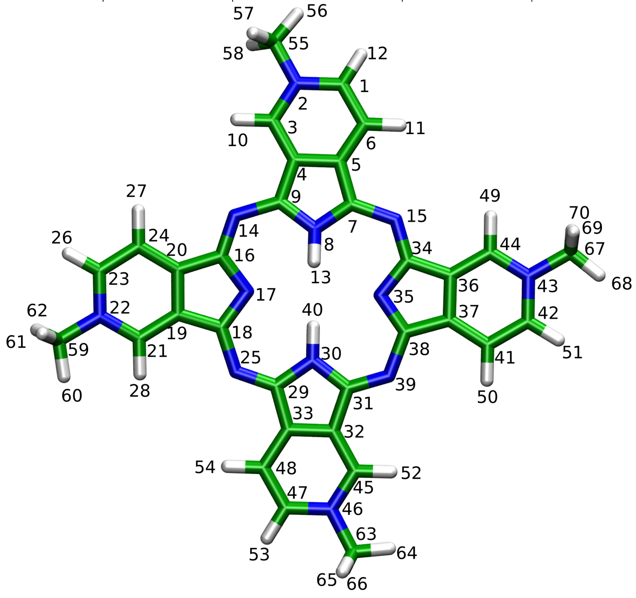

Supplement: S1 Table — (PDF) [file pcbi.1007383.s021.pdf]
