# Supplementary material for: Why do G-quadruplexes dimerize through the 5’-ends? Driving forces for G4 DNA dimerization examined in atomic detail
Source: PLoS Comput Biol. 2019 Sep 20;15(9):e1007383. doi: 10.1371/journal.pcbi.1007383 (PMC6774569; doi:10.1371/journal.pcbi.1007383)
Supplement: Attachment — Submitted filename: Review-G4-dimerization-PLOS-COMPBIOL-07-2019.pdf [file pcbi.1007383.s022.pdf]

The authors applied explicit solvent simulation and free energy calculation to characterize the dimerization of G-quadruplexes and how it can be modulated by ligand binding and ionic concentration. The simulation appears to have been done with care, and the research topic itself , the aggregation of G4 is an interesting and important subject. Therefore, it might be of potential interest to the readers of the PLOS Comp. Biol.

However, I have several concerns that need to be addressed before I can recommend the acceptance for the publication of the manuscript at the PLOS Comp. Biol.

1. During the umbrella sampling simulation to compute the free energy profile of association, is the relative orientation of the two G-quadruplex monomers restrained? It is hard to imagine the umbrella sampling can converge without some kind of orientation restraints, even with microsecond simulations.
2. Page 4, the section header states that the G4 dimerize exclusively via direct guanine-guanine contact, yet according to Fig. 3A, the guanine-guanine interaction energy is zero. How to explain and reconcile the two observations?
3. Page 10, the formula of standard free energy of association is presented without proof. A proof is required if it talks about standard association free energy. For an example of a rigorous derivation of standard association free energy using the PMF approach, see Deng et al. J. Phys. Chem. B, 2017, 121, 10484.
4. Page 3 line 56. It is said that berberines favors 3'end binding and cited papers on crystal structures. However, in solution, it is found that berberines binds with 5'-end and induces extensive reorganization at the 5'-end. See Lin et al. Angew Chem Int Ed Engl, 2018, 57: 10888. Clearly, there is difference between the crystal environment and the solution, and I believe the latter is more relevant biologically.
5. The study used CHARMM36 and amber bsc1 force fields. However, recent studies show that the parmbsc1 force field can lead to distortions of the 5'-end region, compared with the relatively old parmbsc0, which seemed to behave somewhat better. See Deng et al. Molecules, 2019, 24, 1574.
