# Supplementary material for: Why do G-quadruplexes dimerize through the 5’-ends? Driving forces for G4 DNA dimerization examined in atomic detail
Source: PLoS Comput Biol. 2019 Sep 20;15(9):e1007383. doi: 10.1371/journal.pcbi.1007383 (PMC6774569; doi:10.1371/journal.pcbi.1007383)
Supplement: Attachment — Submitted filename: response_to_reviewers.pdf [file pcbi.1007383.s023.pdf]

We thank the reviewers for their valuable suggestions and insightful comments that helped improve the quality of our manuscript. Below, we address each of the points raised by the reviewers.

Referee: 1

In this work the authors studied the stacking patterns of two G-quadruplexes using large-scale replica exchange MD simulations. Based on the simulations, they gave the dominant stacking model, analyzed the corresponding structures in detail, and investigated the underlying interactions for the stability. They also studied the binding free energies as a function of salt concentrations. The knowledge gained in the study may facilitate controlling G4 assembling and designing new G4 oligomers. Overall, I found the simulations were well done, the analysis was sufficient, and the results were solid. I suggest with acceptance of the paper for publication, provided the following minor problems are fixed.

1) Figure captions are not complete, e.g., what are the shaded regions in Fig. 1, what are the color codes in Fig. 2 and 4?

The figure captions have been fixed as recommended by adding color information.

2) In page 9, line 375, the author mentioned with 42 K+ counterions The sentence is hard to understand. And what the equivalent concentration of this with 42 K+ counterions?

The sentence have been fixed and the (estimated) lower salt concentration is now stated explicitly in the text (page 9, lines 387–389).

3) There are some relevant works published previously, e.g., Y. Bian, et al., PLOS CB 2014, v10, e1003562, and J. Zhou, et al., JACS 2017, v139, 7768, the author may want to discuss their relevance with this work.

We thank the reviewer for bringing these interesting works to our attention. Since the first one is particularly relevant for the discussion of the role of ions in G4 stability and interactions, it is now cited in the Introduction (p. 2, l. 11).

Referee: 2

The paper by Czub et al. is a computational study of preferential orientations of GQ dimerization process. From the well-converged free energy calculation as a function of a distance between two guanine cores, they found that G-quadruplexes preferentially dimerize through the 5'-ends. The reason for this 5'-end preference is due to fine balance between interphosphate repulsion and counterion binding. This paper is well written and demonstrates a power of molecular dynamics simulation methods. I would recommend this paper for the publication of PLOS Comp. Biol. after minor revisions.

1) Although the authors pointed out an important role of the enthalpic contribution for such dimerization process and decomposed the enthalpic contributions into several factors, it is difficult to find the total enthalpic and entropic changes in the manuscript. These total enthalpic and entropic changes need to be specified at the first place to clearly state whether the dimerization is due to enthalpic or entropic origin.

As recommended by the reviewer, in the revised manuscript, we discuss the total dimerization enthalpies. The obtained large positive enthalpies suggest that the main driving force for dimerization of like-charged G4 monomers is of entropic origin, which is now explicitly stated in the text (p. 5, l. 188–191, 196–198).

2) The authors concluded that 5'-end binding is preferred mainly due to less interphosphate repulsion. Are there any indications of sugar puckering changes for 5'-end, 5-3 end, 3-end dimerization cases to modulate such interphosphate repulsive interactions?

We agree that changes in sugar puckering might affect the intramolecular distances between phosphate groups, which could in fact be important for the adaptation of the G4 structure at the dimer interface. Therefore, we have now analyzed the changes in sugar puckering upon dimerization, as recommended. We found that dimerization causes only a small decrease in the equilibrium population of C1'-exo puckering in favor of other conformations. Moreover, all dimerization modes differ only slightly in terms of sugar puckering, which cannot fully explain the substantial differences in interphosphate distances across the interfaces. The appropriate discussion and relevant figure are included in the revised manuscript (p. 5, l. 166–168, S10 Fig).

3) In the dimerization via 5'-end, 5-3 end, or 3-end, are there any distinctive perturbation of loop regions?

The cluster analyzes presented in the original manuscript have already shown that direct dimerization through the 5'-interfaces prevents stacking of the loop adenines on the 5'-terminal G-tetrads that is typically observed for monomeric parallel-stranded G-quadruplexes (see Fig. 2 and S5 Fig). In addition, in the revised version, we compared the range of backbone fluctuations in the three dimerization modes with that in the monomer and found that in each case the fluctuations are very similar (S11 Fig).

4) In the GQ dimer, spatial distribution of potassium ions was plotted. In comparison with ion distribution, it would be nice to plot spatial distribution of water at the binding interface to gauge any possible dehydration effect after the dimerization.

Following this suggestion, we carried out additional analyzes aimed at visualizing differences in the spatial distribution of water at the dimerization interfaces as well as quantifying dehydration resulting from the formation of G4 dimers (see S18 Fig.). We found that the interfacial phosphates in the 3-3 mode are somewhat less hydrated compared to the monomeric state (RDF plot in the S18 Fig), but in general the differences in hydration of the three interfaces are not as pronounced. This has been briefly discussed in the revised manuscript (p. 7, l. 298–301).

5) For 3,4-TMPyPz ligand, which was used for the 3-3 dimer, the partial charges they derived need to be included in SI.

The partial charges are now provided in the revised SI (S1 Table).

Referee: 3

The authors applied explicit solvent simulation and free energy calculation to characterize the dimerization of G-quadruplexes and how it can be modulated by ligand binding and ionic concentration. The simulation appears to have been done with care, and the research topic itself, the aggregation of G4 is an interesting and important subject. Therefore, it might be of potential interest to the readers of the PLOS Comp. Biol. However, I have several concerns that need to be addressed before I can recommend the acceptance for the publication of the manuscript at the PLOS Comp. Biol.

1) During the umbrella sampling simulation to compute the free energy profile of association, is the relative orientation of the two G-quadruplex monomers restrained? It is hard to imagine the umbrella sampling can converge without some kind of orientation restraints, even with microsecond simulations.

This is a very good point. In fact, we have not used any orientational restraints, which is now

explicitly stated in the text for clarity (p. 10, l. 435–436). Even without such restraints our profiles seem to be well-converged with a reasonably low statistical uncertainty (see Fig. 1 and S2 Fig). We believe that this results from a careful preparation of the initial structures and applying the replica exchange scheme. Nevertheless, to examine the sampled space of relative orientations and thus better explain the relatively fast free energy convergence, in the revised manuscript, we have included maps describing how the the relative orientation of G4 units depends on the distance between them (see added S1 Fig). The maps confirm that G-quadruplexes dimerize through cofacial stacking (parallel orientation of the interacting G-tetrads at short distances) but also show that at longer distances the systems sample the entire range of relative orientations between G-quadruplexes already on the microsecond timescale, possibly facilitating the free energy convergence in our umbrella sampling simulations. This is also briefly discussed in the text (p. 3, l. 91–94)

2) Page 4, the section header states that the G4 dimerize exclusively via direct guanine-guanine contact, yet according to Fig. 3A, the guanine-guanine interaction energy is zero. How to explain and reconcile the two observations?

The guanine-guanine interaction energies are indeed much smaller than the dominant contributions, such as the repulsion energy between phosphates, and thus they are not as apparent despite the logarithmic scale. That is why numerical values of all interaction energies are also provided in S13 Fig. Additionally, we explicitly refer to the guanine-guanine term in the text (p. 6, l. 246–249) stating that these interactions favor dimer formation ($\Delta H_{\text{G1-G2}} \approx -34$–$40\,\text{kcal/mol}$ in the G-mediated case.

3) Page 10, the formula of standard free energy of association is presented without proof. A proof is required if it talks about standard association free energy. For an example of a rigorous derivation of standard association free energy using the PMF approach, see Deng et al. J. Phys. Chem. B, 2017, 121, 10484.

As it has been already mentioned, we did not used any additional restraints in our REUS simulations, so the formula for standard binding free energy in our case reduces to a simple familiar form. This is now explicitly stated in the text and relevant references have been added for clarity (p. 10, l. 440–444).

4) Page 3 line 56. It is said that berberines favors 3'-end binding and cited papers on crystal structures. However, in solution, it is found that berberines binds with 5-end and induces extensive reorganization at the 5-end. See Lin et al. Angew Chem Int Ed Engl, 2018, 57: 10888. Clearly, there is difference between the crystal environment and the solution, and I believe the latter is more relevant biologically.

We agree with the reviewer that the crystal environment may favor a different binding mode than the one dominant in solution, as is probably also the case for berberine binding to G-quadruplexes. We have modified the cited papers accordingly by referring to berberines without suggesting a particular binding mode. (p. 3, l. 55,58,59,61).

5) The study used CHARMM36 and amber bsc1 force fields. However, recent studies show that the parmbsc1 force field can lead to distortions of the 5'-end region, compared with the relatively old parmbsc0, which seemed to behave somewhat better. See Deng et al. Molecules, 2019, 24, 1574.

Since the most appropriate methodology for simulating G-quadruplexes is still a matter of debate, we decided to use two different force fields that have been reported to yield satisfactory results. In particular, we chose the Charmm36 force field based on a recent study of Salsbury and Lemkul (J. Phys. Chem. B 123, 148-159, 2019) showing that this force field provides a reasonable description of the stability and dynamics of G-quadruplexes. The choice of the Amber bsc1 force field was motivated by a very good agreement of simulated and experimental structures reported in the original study by the Orozco group (Nat. Methods 13(1), 55-58, 2016). Importantly, the results obtained with both

force fields are generally consistent, which we believe corroborates our interpretations and conclusions. We also did not observe any considerable distortions of the 5'-end regions.
