# Supplementary material for: Why do G-quadruplexes dimerize through the 5’-ends? Driving forces for G4 DNA dimerization examined in atomic detail
Source: PLoS Comput Biol. 2019 Sep 20;15(9):e1007383. doi: 10.1371/journal.pcbi.1007383 (PMC6774569; doi:10.1371/journal.pcbi.1007383)
Supplement: Attachment — Submitted filename: Review-G4-dimerization-PLOS-COMPBIOL-08-2019-revised.pdf [file pcbi.1007383.s024.pdf]

The authors have addressed most of the concerns I have raised in the previous round of review, and the manuscript is substantially improved. However, there is one remaining concern that needs to be addressed before it can be accepted:

On page 10, line 441, the formula for the standard dimerization free energy is still not correct. The factor within the logarithm, $\frac{1}{V_o}\int_0^R \exp(-\frac{G(r)}{kT})dr$, is not dimensionless, which is incorrect. The reason is that both the translational and rotational entropy losses upon dimerization are missing from the formula. The authors should add a factor related to the range of the lateral motion of the center of masses of the two G4 monomers in the bound state. In addition, the rotational entropy loss upon dimerization also needs to be added to the formula. The complete formula should be something like

$$\Delta G_{dim}^{o} = -kT \ln \frac{<\Delta\xi_1{}^2>^{\frac{1}{2}}<\Delta\xi_2{}^2>^{\frac{1}{2}}<\Delta\xi_3{}^2>^{\frac{1}{2}}}{8\pi^2} \frac{<\Delta x^2>^{\frac{1}{2}}<\Delta y>^{\frac{1}{2}}}{V_o} \int_0^R \exp(-\frac{G(R)}{kT})dr$$

See Swanson et al. Biophys. J., 2004, pp 67-74 for a detailed description on how to properly estimate the translational and rotational entropy losses upon binding.

Alternatively, it may be possible to just focus on the potential of mean force G(r) computed from the umbrella sampling simulation, and use G(r) as the basis to distinguish the stabilities of the different dimerization modes, with the assumption that the translational and rotational entropy losses upon dimerization are the same for all three binding modes, i.e. 3-3, 3-5, 5-5. This might be a severe approximation though.
