# Supplementary material for: Why do G-quadruplexes dimerize through the 5’-ends? Driving forces for G4 DNA dimerization examined in atomic detail
Source: PLoS Comput Biol. 2019 Sep 20;15(9):e1007383. doi: 10.1371/journal.pcbi.1007383 (PMC6774569; doi:10.1371/journal.pcbi.1007383)
Supplement: Attachment — Submitted filename: response_to_reviewer.pdf [file pcbi.1007383.s025.pdf]

We thank the reviewer for pointing out some inaccuracies in our treatment.

Referee:

The authors have addressed most of the concerns I have raised in the previous round of review, and the manuscript is substantially improved. However, there is one remaining concern that needs to be addressed before it can be accepted:

On page 10, line 441, the formula for the standard dimerization free energy is still not correct. The factor within the logarithm, $\frac{1}{V_0}\int_0^R \exp(-\frac{G(r)}{kT})$, is not dimensionless, which is incorrect. The reason is that both the translational and rotational entropy losses upon dimerization are missing from the formula. The authors should add a factor related to the range of the lateral motion of the center of masses of the two G4 monomers in the bound state. In addition, the rotational entropy loss upon dimerization also needs to be added to the formula. The complete formula should be something like

$$\Delta G^0_{dim} = -kT \ln \frac{\langle \Delta\xi_1^2\rangle^{\frac{1}{2}} \langle \Delta\xi_2^2\rangle^{\frac{1}{2}} \langle \Delta\xi_3^2\rangle^{\frac{1}{2}}}{8\pi^2} \frac{\langle \Delta x^2\rangle^{\frac{1}{2}} \langle \Delta y^2\rangle^{\frac{1}{2}}}{V_0} \int_0^R \exp(-\frac{G(r)}{kT})\mathrm{d}r \quad (1)$$

See Swanson et al. Biophys. J., 2004, pp 67-74 for a detailed description on how to properly estimate the translational and rotational entropy losses upon binding. Alternatively, it may be possible to just focus on the potential of mean force G(r) computed from the umbrella sampling simulation, and use G(r) as the basis to distinguish the stabilities of the different dimerization modes, with the assumption that the translational and rotational entropy losses upon dimerization are the same for all three binding modes, i.e. 3-3, 3-5, 5-5. This might be a severe approximation though.

We agree that rigorous determination of the standard binding free energy would require a careful treatment of translation and rotational degrees of freedom, particularly in the bound state. However, we believe that, given the amount of our simulation data, direct estimation of entropy losses might not be reliable enough (it has been shown repeatedly in the past that free energy converges much faster then entropy and enthalpy alone).

Therefore we decided to follow the alternative reviewer's suggestion and compared the relative stabilities in a semi-quantiative fashion, based entirely on the free energy profiles $G(r)$. We agree that this requires the assumption about a similar loss of translation and rotational entropy upon dimerization to all three modes which is now stated explicitly in the revised manuscript. (p. 3 l. 97–99 and caption of Table 1).

Regarding the dimension issue, in the original formula, the function $G(r)$ is taken directly from the WHAM procedure, without any correction. Thus the corresponding probability density $\rho(r) \propto \exp(-\beta G(r))$ is effectively already integrated over the angular coordinates, assuming the spherical symmetry. For clarity, we modified the formula in the revised manuscript to include the angle intergration explicitly (p. 10–11 l. 440–445).
